# Infected urachal remnant presenting with purulent umbilical discharge in an adult: A diagnostic and surgical challenge

**DOI:** 10.1016/j.eucr.2025.103265

**Published:** 2025-11-05

**Authors:** Quang Anh Dao, Tuan Anh Tran, Dinh Thanh Son Le, Thanh Tung Tran

**Affiliations:** a Department of Diagnostic Imaging, Phu Tho Province General Hospital, Phu Tho, Viet Nam; b Department of Urology, Phu Tho Province General Hospital, Phu Tho, 290000, Viet Nam; c Department of General Surgery, Phu Tho Province General Hospital, Phu Tho, 290000, Viet Nam

**Keywords:** Infected urachal cyst, Adult urachal anomaly, Umbilical discharge, Laparoscopic urachectomy

## Abstract

Urachal anomalies are rare in adults and may mimic common abdominal conditions, leading to delayed or missed diagnosis. We report a 47-year-old man presenting with isolated purulent umbilical discharge initially mistaken for a superficial abscess. Contrast-enhanced computed tomography revealed a rim-enhancing lesion with a non-communicating urachal tract. The patient underwent successful laparoscopic excision, and histopathology confirmed an infected urachal remnant without malignancy. Recovery was uneventful, with no recurrence at one month. This case emphasizes the diagnostic value of early imaging and supports laparoscopic urachectomy as a safe, definitive management for infected adult urachal remnants.

## 1 Introduction

The urachus is a vestigial remnant of the embryonic allantoic duct, which typically involutes prior to birth to form the fibrous median umbilical ligament extending from the bladder dome to the umbilicus.^1^^,^^2^ Failure of complete obliteration results in persistent urachal anomalies, classically categorized into four types: patent urachus, urachal sinus, urachal diverticulum, and urachal cyst. Among these, urachal cyst is the most commonly observed anomaly in adults.^3^^,^^4^

Although these anomalies are usually identified during infancy or early childhood, they may remain clinically silent until adulthood, where they are often diagnosed incidentally or upon development of complications.^5^ In adults, urachal anomalies are **rare** and frequently detected **incidentally on cross-sectional imaging**.^2^^,^^6^ Symptomatic urachal cysts **constitute a notable subset** of urachal anomalies in adults.^2^

Infected urachal remnants can present with non-specific symptoms such as vague lower abdominal or periumbilical pain, malodorous umbilical discharge, or a tender subumbilical mass.^2^^,^^7^, ^8^, ^9^, ^10^ These findings often mimic a range of common abdominal pathologies, including umbilical hernia, omphalitis, epidermoid cysts, or infected umbilical granulomas or urachal sinuses, thereby increasing the risk of misdiagnosis or delayed recognition.^2^^,^^7^^,^^8^^,^^10^ In particular, the overlapping clinical presentation with superficial abscesses or incarcerated umbilical hernia may lead to initial conservative or inappropriate surgical management, highlighting the need for imaging-based confirmation and accurate anatomical delineation.^2^^,^^7^^,^^10^

Cross-sectional imaging plays a pivotal role in establishing the diagnosis and evaluating the extent of disease.^3^^,^^6^ Ultrasonography offers a readily available initial modality but may be limited by operator dependency and anatomical obscuration.^11^ In contrast, contrast-enhanced computed tomography (CT) provides superior delineation of lesion morphology, extent of infection, and potential communication with the urinary bladder or adjacent structures.^3^^,^^6^ CT imaging is particularly valuable in surgical planning and in excluding urachal adenocarcinoma, a rare but potentially fatal complication in recurrent or chronic cases.^3^^,^^6^^,^^12^

Historically, open surgical excision has been the standard treatment. However, laparoscopic urachectomy has gained increasing acceptance as a safe and effective alternative for both uncomplicated and infected urachal remnants, offering advantages such as reduced postoperative pain, shorter hospitalization, and faster recovery.^13^^,^^14^ Nevertheless, reports focusing on laparoscopic management in the context of infected urachal remnants remain limited, particularly in adults whose clinical presentation deviates from classic textbook features.^13^^,^^15^

In this report, we present a rare adult case of an infected urachal cyst initially misdiagnosed as a superficial umbilical abscess. The case underscores the diagnostic challenge of urachal anomalies in adulthood and highlights the critical role of advanced imaging and minimally invasive surgery in achieving definitive and effective treatment.

## 2 Case report

A 47-year-old previously healthy male, working as a secondary school teacher, presented to our surgical department with a 10-day history of progressive periumbilical discomfort accompanied by purulent umbilical discharge. The symptoms developed insidiously and were not associated with fever, nausea, vomiting, dysuria, hematuria, or bowel disturbances. The patient denied any history of prior abdominal trauma, abdominal surgery, or systemic illness.

On physical examination, he was afebrile, alert, and hemodynamically stable. Local inspection of the abdomen revealed a tender, erythematous, and fluctuant mass centered at the umbilicus, with malodorous purulent discharge emerging from a protruding central nodule approximately 1 cm in diameter. The remainder of the abdominal examination was unremarkable, with no signs of peritonitis, organomegaly, or palpable masses. Cardiopulmonary auscultation was normal, except for coarse breath sounds at the lung bases. Based on the localized findings and absence of systemic features, a presumptive diagnosis of superficial umbilical abscess was made.

Laboratory testing revealed mild leukocytosis (WBC 6.65 G/L; neutrophils 60.4 %). Renal and liver function tests, serum electrolytes, and coagulation profiles were within normal ranges. Urinalysis was negative for hematuria or pyuria. Viral markers for HIV, HBsAg, and HCV were non-reactive.

Initial ultrasonography demonstrated a well-circumscribed, hypoechoic lesion measuring 6 × 10 mm within the subumbilical soft tissue, containing anechoic fluid. A midline tubular structure approximately 2 mm in diameter was also identified between the peritoneum and anterior abdominal wall, suggesting a urachal remnant.

**Fig. 1:**
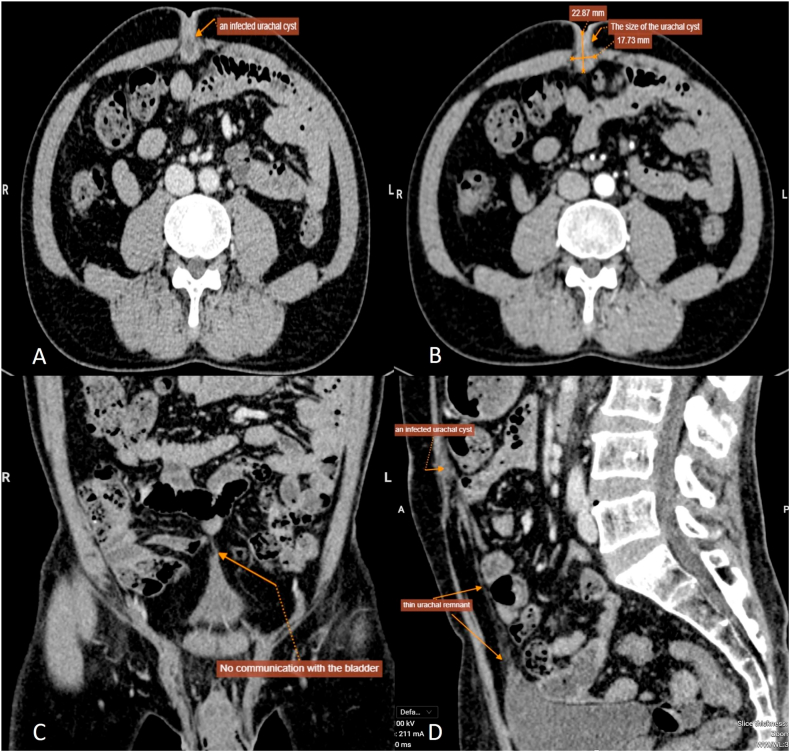
Contrast-enhanced CT imaging of an infected urachal remnant (A) Axial CT scan shows a round, rim-enhancing fluid-filled lesion (22.9 × 17.7 mm) in the midline anterior extraperitoneal space beneath the rectus abdominis, with central hypodensity and mild fat stranding, consistent with an infected urachal cyst. (B) Another axial view confirms confinement of the lesion to the space of Retzius without internal septation or intraperitoneal communication, suggesting a partially obliterated infected urachal remnant. (C) Coronal CT reconstruction illustrates the lesion centrally located between the umbilicus and bladder dome, with no signs of fistulous connection to the bladder. (D) Sagittal view reveals a linear hypodense tract extending caudally toward the bladder dome, representing a remnant urachal tract. No adjacent organ involvement or intraperitoneal fluid is observed.

CT was subsequently performed for further evaluation. It revealed a well-defined, rim-enhancing fluid collection measuring 22.9 × 17.7 mm within the extraperitoneal prevesical space (space of Retzius), accompanied by a linear hypodense tract extending caudally toward the bladder dome. No septations, intraperitoneal fluid, or bowel-related pathology were observed. These findings were consistent with an infected urachal cyst with a partially obliterated tract (Fig. 1).

Based on imaging and clinical presentation, laparoscopic transabdominal excision was performed under general anesthesia using a standard three-port technique. Intraoperatively, a cystic lesion was identified in the preperitoneal space, adherent to the posterior rectus sheath and surrounding fascia. The cyst and entire urachal tract were excised en bloc along with adjacent fibrotic tissue. No definitive communication with the bladder was noted, although limited debridement of the bladder dome was performed prophylactically. The laparoscopic approach provided excellent visualization and minimized surgical morbidity.

**Fig. 2:**
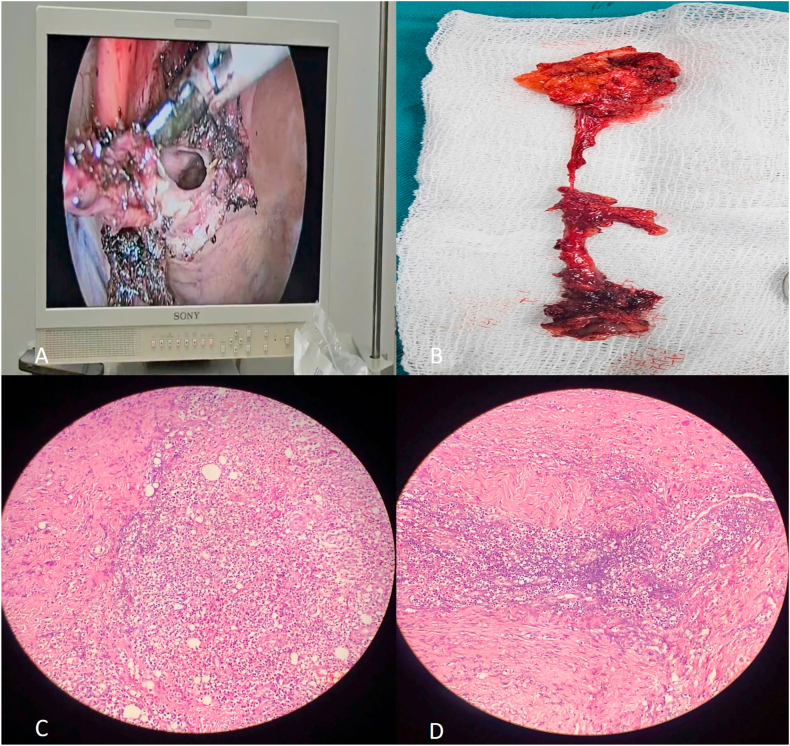
Intraoperative and histopathological findings of an infected urachal remnant (A) Laparoscopic image shows electrocautery-assisted dissection of the infected urachal remnant using a standard three-port transabdominal approach. (B) Gross specimen depicts a tubular urachal remnant with adjacent inflamed fibrotic tissue and signs of cystic degeneration. (C) Histopathological slide at 100 × magnification displays extensive neutrophilic infiltration and necrotic debris within fibrotic tissue, confirming suppurative inflammation. (D) Higher magnification (200 × ) highlights mixed inflammatory infiltrate of neutrophils, lymphocytes, and plasma cells surrounding residual epithelial lining, consistent with an infected urachal remnant without malignancy.

Gross examination showed a tubular specimen with cystic degeneration and inflamed surrounding tissues. Histopathological analysis confirmed an infected urachal remnant, consisting of dense fibrous tissue, focal necrosis, and suppurative inflammation. Microscopy demonstrated residual epithelial lining with prominent neutrophilic infiltration. No dysplasia or malignancy was identified (Fig. 2).

Postoperative recovery was uneventful. The patient received therapeutic antibiotics for infection control rather than routine perioperative prophylaxis. Intravenous cefuroxime 1 g twice daily was given for 24 hours before surgery to control localized infection. Postoperatively, he received ofloxacin 200 mg IV every 12 hours together with meropenem 1 g IV every 12 hours for 7 days, followed by oral levofloxacin 500 mg daily for another 7 days after discharge. This regimen, guided by the initial elevation of C-reactive protein (9.7 mg/L), achieved complete infection resolution without postoperative complications. By postoperative day (POD) 2, the patient remained afebrile, mobile, and tolerating oral intake. Laboratory parameters normalized. A urinary catheter placed intraoperatively was removed on POD 5 following routine bladder irrigation. By POD 8, the patient had resumed full ambulation and was discharged on POD 9 in stable condition.

At the one-month follow-up, the patient remained asymptomatic. Follow-up ultrasonography demonstrated no residual fluid collection or recurrent tract formation. The surgical site had healed appropriately, with no urinary or wound-related complications observed.

## 3 Discussion

Urachal anomalies in adults are rare and typically discovered incidentally or when complicated by infection or, less frequently, malignant transformation.^2^^,^^6^ Among these entities, urachal cysts are the most frequent but often remain clinically silent until acute infection develops.^2^^,^^7^ Prior reports have described infected cysts presenting with periumbilical pain, abdominal swelling, or systemic manifestations such as fever and leukocytosis.^2^^,^^3^^,^^6^^,^^7^ In contrast, the present case was remarkable for its exclusive manifestation of purulent umbilical discharge in the absence of systemic illness, which represents an atypical and clinically misleading profile that initially suggested a superficial abscess. This unusual presentation underscores the diagnostic challenge and differentiates our case from previously published series.

Misdiagnosis is a recurrent issue in both reviews and case reports. Infected urachal cysts have been mistaken for incarcerated hernia, omphalitis, or epidermoid cysts.^2^^,^^7^^,^^9^ Wilson et al. emphasized that the nonspecific nature of umbilical discharge often leads to diagnostic uncertainty,^2^ while Corsello et al. reported a patient initially diagnosed with umbilical hernia,^9^ and Sukiman et al. described an abscess-forming cyst requiring staged drainage before definitive excision.^7^ Unlike these cases, our patient exhibited no systemic inflammatory response and the lesion was small in size, yet it still mimicked a benign superficial abscess. This reinforces the importance of early cross-sectional imaging in characterizing atypical lesions and preventing inappropriate or delayed management.^2^, ^6^

Surgical excision remains the standard of care for symptomatic urachal remnants, preventing recurrence and reducing the risk of malignant transformation.^12^^,^^16^ While open surgery was historically the gold standard, laparoscopic excision has become increasingly accepted owing to its reduced morbidity and faster recovery.^13^, ^14^, ^15^ Earlier reports by Siow et al. and Araki et al. established its feasibility in symptomatic or recurrent cases,^13^^,^^15^ and Ryan et al. subsequently confirmed favorable outcomes in a larger cohort.^14^ What distinguishes our case is that laparoscopic excision was performed in the acute infectious phase without prior drainage and without conversion to open surgery. The uneventful and rapid postoperative recovery further underscores the safety and efficacy of a direct minimally invasive approach even in infected settings.

Histopathological evaluation is indispensable because chronic urachal remnants may undergo malignant transformation, most commonly to adenocarcinoma.^10^^,^^12^^,^^17^ Wilson et al. and Gleason et al. have emphasized the need for histologic confirmation, especially in older adults.^2^^,^^10^ In our case, the patient was 47 years old and therefore within the demographic at increased oncologic risk, yet histology confirmed benign inflammatory changes without dysplasia or carcinoma. This finding validates the oncological safety of complete excision and supports its recommendation for all adult cases, even when preoperative suspicion of malignancy is low.

Finally, urachal anomalies have been documented across diverse populations, including a recent report from Ghana that described a cystic lesion presenting as an abdominal mass.^18^ In contrast to these published cases, our report contributes novelty in three aspects: first, an atypical presentation limited to isolated umbilical discharge; second, the demonstration that immediate single-stage laparoscopic excision is feasible and safe in the acute infection phase without prior drainage; and third, documentation of an uneventful and rapid recovery. Collectively, these features expand the recognized clinical spectrum of infected urachal anomalies and strengthen the evidence base supportingminimally invasive surgery as a definitive and oncologically sound strategy in adult patients.

## 4 Conclusion

This case underscores the importance of considering urachal anomalies in adults presenting with atypical umbilical discharge. Contrast-enhanced CT was pivotal for accurate diagnosis and surgical planning, while laparoscopic urachectomy proved safe and effective. Histopathology confirmed a benign lesion, supporting complete excision as the definitive strategy to prevent recurrence and rule out malignancy.

## CRediT authorship contribution statement

**Quang Anh Dao:** Writing – review & editing, Writing – original draft, Methodology, Investigation, Data curation, Conceptualization. **Tuan Anh Tran:** Writing – review & editing, Investigation, Data curation. **Dinh Thanh Son Le:** Writing – review & editing, Supervision, Resources, Project administration. **Thanh Tung Tran:** Writing – review & editing, Investigation, Data curation.

## Patient consent statement

Written informed consent was obtained from the patient for the publication of this case report and any accompanying images. The patient was fully informed about the nature of the report, its scientific purpose, and the potential for online open-access publication. A copy of the signed consent form is available for review by the Editor-in-Chief of this journal upon request.

## Research registration

Not applicable.

## Ethical approval

Not applicable.

## Guarantor

Dr. Quang Anh Dao accepts full responsibility for the work and the conduct of the study, had access to the data, and controlled the decision to publish.

## Provenance and peer review

This article was not commissioned and was peer-reviewed.

## Funding

This work did not receive any specific grant from funding agencies in the public, commercial, or not-for-profit sectors.

## Declaration of interest statement

The authors declare that they have no known financial or personal relationships that could have appeared to influence the work reported in this case report. No competing interests exist.

## Abbreviations

CT: Contrast-enhanced computed tomography
POD: Postoperative day
